# Resolution Improvement of Light Field Imaging via a Nematic Liquid Crystal Microlens with Added Multi-Walled Carbon Nanotubes

**DOI:** 10.3390/s20195557

**Published:** 2020-09-28

**Authors:** Hui Li, Yi Yu, Jing Peng, Yuntao Wu, Yanduo Zhang

**Affiliations:** 1 School of Computer Science and Engineering, Wuhan Institute of Technology, Wuhan 430205, China; yuyikaeun@163.com (Y.Y.); JingPeng124@gmail.com (J.P.); zhangyanduo@hotmail.com (Y.Z.); 2 Hubei Key Laboratory of Intelligent Robot, Wuhan 430205, China; 3 School of Chemistry and Chemical Engineering, Huazhong University of Science and Technology, Wuhan 430074, China

**Keywords:** liquid crystal microlens, light field imaging, carbon nanotubes, doping

## Abstract

A relatively simple method to improve the image resolution of light field based on a liquid crystal (LC) microlens doped with multi-walled carbon nanotubes (MWCNTs) was developed and evaluated. As the nanoparticles were doped in LC, its electro-optical features could enhance, leading to a short response time compared to the pure LC microlens. With the maximum use of the proposed LC microlens, a method combining aperiodicity extraction and weighted average algorithm was adopted to realize the high-resolution light field imaging. The aperiodicity extraction method was proposed, which could effectively improve resolution of view angle image. For synthesizing the full resolution image at 0 Vrms and the extracted view angle image of light field imaging at 2.0 Vrms, the final high-resolution light field imaging could be obtained in a short time by weighted average algorithm. In this way, the common problem of low resolution in light field imaging could be solved. This proposed method was in good agreement with our experimental results. And it was also in line with the development of the trend of the smart imaging sensor combining algorithm with hardware.

## 1. Introduction

Light field imaging can be seen as next generation imaging technology, which has already attracted much attentions from the whole of the world [1]. It is based on computational imaging theory. And it can effectively solve several serious issues of the conventional imaging systems, such as defocus of scene and depth loss of target object [2,3,4]. Then, it has great potential for adverse condition imaging, depth estimation, target focusing, and foreground removal [5,6,7,8]. In light field imaging, the extraction of view angle image is the foundation [9]. The resolution of view angle image will directly affect the accuracy of subsequent calculation in applications, such as depth estimation and stereo matching [10]. Therefore, the extraction is a key factor for improving the resolution of light field imaging. In this field, there are many different kinds of methods. Usually, they can be divided into two classes. Some studies have focused on the layout of optical glass microlens array, such as moving lens array technique [11,12]. And the other research hotspots are the extraction of pixel points [13,14]. Typically, the common method is the periodicity extraction algorithm, which is the most easy and fast mainstream approach. But, this method does not consider the angle of the incident light. In this way, it will lead to aliasing, distortion, low accuracy, and low spatial resolution issues. For the low-resolution problem, some interpolation algorithms have been proposed, such as bilinear interpolation and adaptive projection iteration. However, those algorithms are all based on the known pixels to estimate interpolation points. Its authenticity is not very high. With the rapid development of neural network and deep learning, a large number of algorithms about sparse dictionary learning and convolution neural network have emerged in recent years. But, these approaches still need a large amount of data and long training time, which would limit its application range. To improve the resolution, an antagonism network with a single view angle image has been proposed [15]. This kind of algorithm has good fitting effect. However, it still uses similar interpolation fitting method, which will directly cause data error. In addition, the majority of researches about light field imaging systems mentioned above have always used the conventional optical glass microlens array as the core hardware platform. There are few studies about several non-glass kinds of optical elements in light field imaging, which are worthy to do more in-depth and detailed study. Generally speaking, those traditional optical microlens is very easy to be restricted by aperture, depth of field, exposure time, and exposure level, which could seriously restrict the imaging performance of the traditional glass microlens [16]. And those problems are unavoidable in affecting the effect of light field imaging. Therefore, a new kind of optical imaging element is urgently explored in this field. And the adaptive methods matching the new optical element, such as the view angle image extraction algorithm, are also urgently studied.

Liquid crystal (LC) is a classic electro-optical material. It has been widely studied by many scientists from various countries. A variety of research studies on it have been developed in recent decades. The effective LC birefringence can be controlled by an external electric field. Thus, the electro-optical features of LC can be also tunable by the external electric field. Depending on the molecular alignment, several useful LC devices have been developed. The well-known is a gradient refractive index for adaptive focus lenses. For imaging applications, homogeneous-aligned LC offers a possibility to image without any mechanical movements. The main advantages are voltage actuation, low power consumption, simple fabrication, compact structure, and good stability [17,18,19,20]. In past decades, tunable-focus LC lenses have been studied. They have been widely applied in many fields, especially in 3D display [21,22,23,24]. In recent years, there have been some researches for applying LC microlens in light field imaging, mainly focused on extending depth of field [25,26,27,28]. However, the studies on improvement of resolution for light field imaging via LC microlens are not enough, which are worthy of further study.

In view of the above-mentioned problems, based on LC microlens sampling principle and geometrical optics, an aperiodicity algorithm for LC microlens has been proposed to extract high resolution view angle image, which can not only take into account in the direction information of light but also extract the view angle image with higher data accuracy. By reducing appropriate small view angle range, the pixel block of the projection area of LC microlens is extracted, which can ensure the accuracy of the data and improve the resolution of view angle image at the same time. In addition, the electrically tunable switching feature of LC microlens has been applied to improve the resolution of light field imaging. With the use of weighted average algorithm, two different light field data at various voltages can be utilized to generate the high quality light field imaging. To realize this aim, a LC microlens with fast response time is urgently needed. There are also many methods to fabricate high performance LC lens, especially on fast response time. In this study, the nano-doping technology has been chosen [16,29,30,31,32]. Compared to the conventional method of constructing new structure, the proposed nano-doping method has relatively significant advantages in enhancing the performances of LC microlens with the compromise of many factors in light field imaging [17,18,19,20]. In this way, light field imaging via using a nematic LC microlens added with multi-walled carbon nanotubes (MWCNTs) is presented. With the maximum use of the LC microlens, an adaptive method combining with aperiodicity extraction and weighted average algorithm has been proposed.

## 2. Materials and Methods

### 2.1. Sample Preparation

**Figure 1:**
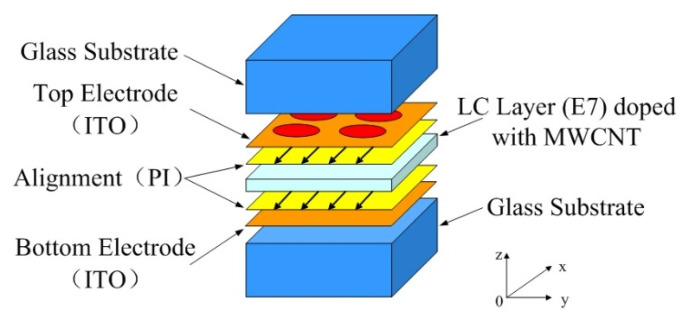
The schematic structure of the liquid crystal (LC) microlens.

The structure of the LC microlens consists of two Indium Tin Oxide (ITO) electrodes, two glass substrates, two alignment layers of polyimide (PI), and LC layer doped with MWCNTs, as shown in Figure 1. The top ITO electrode has been fabricated by a UV-photolithography and a wet-etching procedure for forming an aperture-pattern array with a diameter of 140 μm and a pitch of 140 μm. The number of array in the LC microlens is 128 × 128. The directions of rubbing those alignment layers have been all along the *x*-axis. The thickness of LC layer is 20 μm, which is determined by microsphere spacers. E7 LC from Merck Co. (Shanghai, China) has refractive indexes of *n*_e_ = 1.7472 and *n*_o_ = 1.5217 at 20 degree and 589 nm wavelength. In order to improve the response time, MWCNTs from Shenzhen nanotech port Co. Ltd. (Shenzhen, China). has been chosen as a dopant. The reason for not choosing single-walled carbon nanotubes (SWCNTs) is that the diameter of SWCNTs is very small, generally between 1 nm and 2 nm, the specific surface energy is very high, it exists in bundles in most cases, and it is semi-conductor compared to MWCNTs. The amount of MWCNTs is about 0.02 wt.%, and all the experiments have been realized at this value. Preliminary experiments have established the most intriguing concentrations of MWCNTs in LC-MWCNTs mixture [16,32].

The most important step of fabrication is as follows. In order to eliminate agglomeration, the MWCNTs have been firstly grinded by metal power grinder. For twice grinding, every time has lasted 30 s. Then, the nematic LC and MWCNTs have been mixed together. Ultrasound agitator and molecular agitator have been utilize in turn to generate a homogeneous mixture. This mixture after three days later has been poured into LC cell by capillary at 80 degree Celsius. At last, the LC cell has been sealed by AB glues. Previous experiments have established that the ultrasonic vibration and grinding method is so effective that the mixture can be kept in uniform state in around one month, and it has an improvement electro-optical features compared to the pure LCs [16,32].

### 2.2. Light Field Imaging Based on LC Microlens

**Figure 2:**
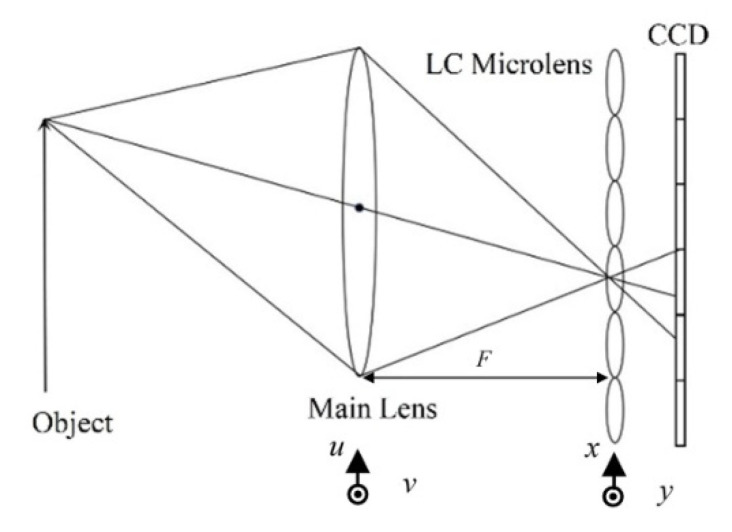
Light field imaging acquisition biplane parameter model based on LC microlens.

As shown in Figure 2, the light field imaging system based on LC microlens is presented. The LC microlens inserts between the main lens and charge coupled device (CCD), so as to record the light direction and light radiation at the same time. The main lens is in front of LC microlens to construct the first imaging subsystem. And the LC microlens and CCD make up a hybrid imaging system as the second imaging subsystem. In this study, the biplane parameter model is chosen as the representation of light field [33].

According to the theory of light field rendering, an image sampling by the biplane parameter model can be expressed as [33]

(1) $$E_{F}\left(x,y\right)=\frac{1}{F^{2}}\iint L_{F}\left(x,y,u,v\right)dudv,$$

where (x,y) is the image plane, (u,v) is the main lens plane, F is the distance between the main lens plane and the image plane, $E_{F}\left(x,y\right)$ represents the image formed on the image plane, and $L_{F}\left(x,y,u,v\right)$ represents a sampling of the light field.

Based on the theory of light field, the light field imaging system can get any two-dimensional (2D) view angle images after one exposure. According to Equation (1), if the integral is calculated, different view angles image at different focusing depths can be got.

In $L_{F}\left(x,y,u,v\right)$, the value range (x,y) of the variables in the light field is determined by the CCD pixel, and the value range (u,v) of the variables is determined by the main lens aperture, as shown in Figure 2. If $u=u^{*}$ and $v=v^{*}$ are fixed, the formula for calculating the image formed by the sub-aperture of the light field is [33]

(2) $$E_{F}\left(x,y\right)=\frac{1}{F^{2}}\int_{u}^{u+\Delta u}\int_{v}^{v+\Delta v}L_{F}\left(x,y,u,v\right)dudv,$$

where $E_{F}\left(x,y\right)$ is the view angle image of viewpoint $\left(u^{*},v^{*}\right)$.

**Figure 3:**
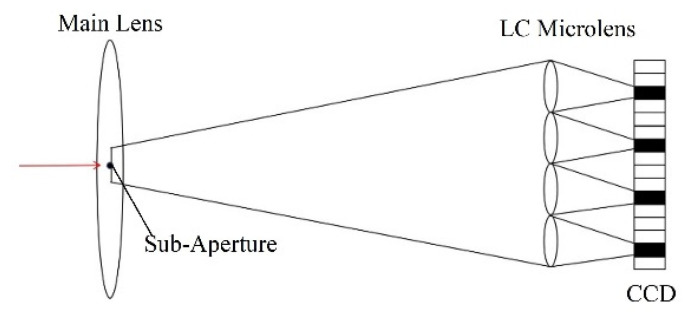
View angle image extraction schematic diagram.

As shown in Figure 3, every sub-microlens of LC microlens corresponds to a small area of CCD pixel. The view angle image is to extract single one pixel from the same position of a small area of CCD corresponding to every sub-microlens of LC microlens. According to their sub-microlens positions, these pixels are rearranged. Thus, a view angle image is formed. It can be seen that the view angle image is equivalent to the image formed by the LC microlens after the main lens reduces the aperture and the resolution of the view angle image is equal to the number of the LC microlens. It can also be found that the light field imaging system based on LC microlens is to alter angle resolution at the cost of spatial resolution. In this way, the view angle image can be seen as a 2D slice of light field $L_{F}\left(x,y,u,v\right)$ in the direction dimension.

For the traditional optical imaging system, light field refocusing is to change the distance between the main lens and the CCD plane. That means changing the image distance can acquire another refocusing state.

**Figure 4:**
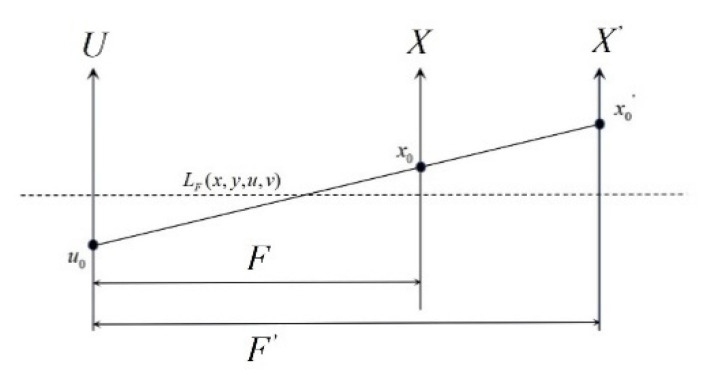
Schematic diagram of light field refocusing. The plane *X* is the first imaging state, and the plane *X*’ is the refocused imaging state.

The schematic diagram of the light field refocusing principle is shown in Figure 4. The distance between the main lens and the image plane is F. Under this situation, the sampled light field is $L_{F}\left(x,y,u,v\right)$. The main lens plane U and the image plane X has the intersection point $u_{0}$ and intersection point $x_{0}$, respectively. If the image distance is changed to $F^{\prime }$, and the light intersects the new image plane $X^{\prime }$ at the point $x_{0}{}^{\prime }$. The image calculation formula of the new image plane can be seen as follows [33]:

(3) $$E_{F^{\prime }}\left(x^{\prime },y^{\prime }\right)=\frac{1}{{F^{\prime }}^{2}}\iint L_{F^{\prime }}\left(x^{\prime },y^{\prime },u,v\right)dudv.$$

Because $L_{F}\left(x,y,u,v\right)$ and $L_{F^{\prime }}\left(x^{\prime },y^{\prime },u,v\right)$ are the same light,

(4) $$L_{F^{\prime }}\left(x^{\prime },y^{\prime },u,v\right)=L_{F}\left(x,y,u,v\right).$$

According to the principle of similar triangle,

(5) $$\frac{x-u}{x^{\prime }-u}=\frac{F}{F^{\prime }},$$

and

(6) $$x=u+\left(x^{\prime }-u\right)\frac{F}{F^{\prime }}.$$

By substituting Equation (6) into Equation (3) and Equation (4), it is easy to deduce [33]:

(7) $$\begin{array}{ll} E_{F^{\prime }}\left(x^{\prime },y^{\prime }\right) & =\frac{1}{{F^{\prime }}^{2}}\iint L_{F^{\prime }}\left(x^{\prime },y^{\prime },u,v\right)dudv,\\ & =\frac{1}{{F^{\prime }}^{2}}\iint L_{F}\left(u+\left(x^{\prime }-u\right)\frac{F}{F^{\prime }},v+\left(y^{\prime }-v\right)\frac{F}{F^{\prime }},u,v\right)dudv,\\ & =\frac{1}{{F^{\prime }}^{2}}\iint L_{F}\left(u\left(1-\frac{F}{F^{\prime }}\right)+x^{\prime }\frac{F}{F^{\prime }},v\left(1-\frac{F}{F^{\prime }}\right)+y^{\prime }\frac{F}{F^{\prime }},u,v\right)dudv. \end{array}$$

If $\alpha =\frac{F^{\prime }}{F}$,

(8) $$E_{\alpha F}\left(x^{\prime },y^{\prime }\right)=\frac{1}{{\left(\alpha F\right)}^{2}}\iint L_{F}\left(u\left(1-\frac{1}{\alpha }\right)+\frac{x^{\prime }}{\alpha },v\left(1-\frac{1}{\alpha }\right)+\frac{y^{\prime }}{\alpha },u,v\right)dudv,$$

where $E_{\alpha F}\left(x^{\prime },y^{\prime }\right)$ is the refocused image, and Equation (8) is the calculation formula of refocusing of the light field.

### 2.3. Resolution of Light Field Imaging Based on LC Microlens

**Figure 5:**
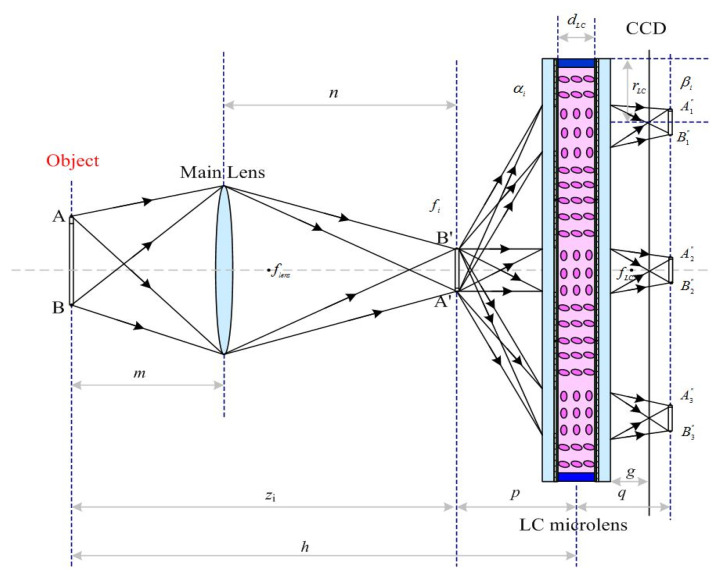
The schematic imaging system setup of the light field imaging based on LC microlens.

The schematic imaging system setup is as shown in Figure 5. *AB* has been chosen as the object in a scene. After the first imaging, there is a real image *A*’*B*’ behind the main lens. On the CCD, an array of elemental virtual images $A_{1}^{"}B_{1}^{"}$, $A_{2}^{"}B_{2}^{"}$, and $A_{3}^{"}B_{3}^{"}$ can be obtained. In Figure 5, m means the object distance, n denotes the imaging distance, p presents the object distance for second imaging, q is the imaging distance for second imaging, $z_{i}$ is the distance between object and the first imaging, *h* means the distance between object and LC microlens, *d*_LC_ means the thickness of the LC layer, *r*_LC_ represents the radius of the single aperture of the LC microlens, *f*_lens_ is the focal length of the main lens, *f*_LC_ is the focal length of the LC microlens, and *g* refers to the distance between the LC microlens and CCD.

To characterize the view resolution of light field imaging based on LC microlens, the approximate theoretical analysis of view resolution of light field imaging is as follows. The influence of focusing error has been not taken into account. After first imaging, the spatial resolution of the obtained image is assumed as *f*_i_. Then, the spatial resolution of the LC microlens is [34]

(9) $$\alpha_{i}=f_{i}\left|h-z_{i}\right|.$$

Correspondingly, the viewing spatial resolution is [34]

(10) $$\beta_{i}=f_{i}z_{i}=\frac{\alpha_{i}z_{i}}{\left|h-z_{i}\right|}.$$

If the pitch between LC microlens is $\omega_{l}$, the sampling period is $\omega_{l}/h$. Then, Nyquist sampling resolution is [34]

(11) $$\beta_{nyq}=\frac{h}{2\omega_{l}}.$$

When the maximum viewing spatial resolution is above that of Nyquist, the obtained imaged on the image plane of CCD is occluded because of aliasing. Under this condition, the actual view spatial resolution is [34]

(12) $$\beta_{\max}=\min(\beta_{i\max},\beta_{nyq})=\min\left(\frac{\alpha_{i\max}z_{i}}{\left|h-z_{i}\right|},\beta_{nyq}\right).$$

### 2.4. High Resolution of Light Field Imaging Based on LC Microlens

**Figure 6:**
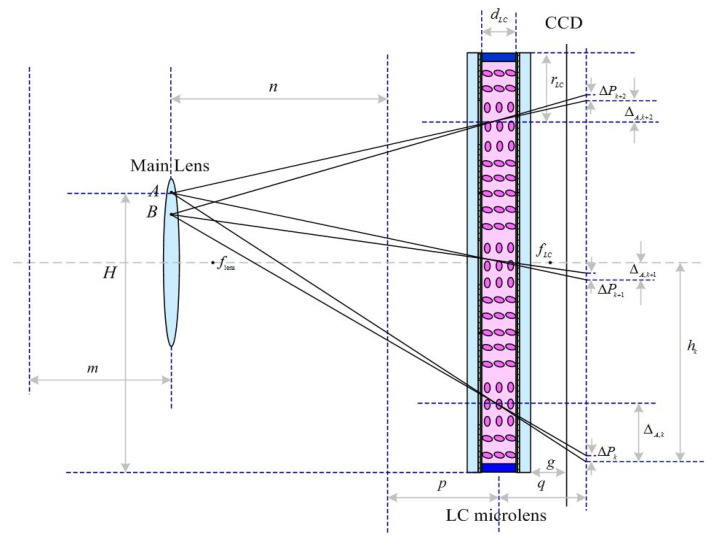
Schematic diagram of aperiodicity extracting the view angle image in light field imaging based on LC microlens.

With the use of geometry optics, the theoretical analysis for high resolution of light field imaging based on LC microlens is as follows. The parameters in Figure 6 are the same as thosementioned in the Section 2.3.

The aperiodicity extraction method for LC microlens is as follows:

(1) Calculating the central coordinates of every microlens

To extract the view angle image, the location of the element image is firstly needed to determine. Specifically, the tilt of the white image is firstly needed to be correct. Then, the element image is segmented according to the corrected tilt white image. Finally, the center values of the element image can be got.

(2) Calculating the migration

When calculating view angle image, it is always assumed that the transmitted light rays are parallel. Thus, when extracting pixels from every microlens, the offset of each microlens is identified as the same. Generally, the periodic extraction method does not taken into account in the direction of light. For any viewpoint, the offset of microlens is actually different. Thus, the pixels in the view angle image should be extracted aperiodicity.

In Figure 6, the viewpoint *A* is on the main lens plane, the distance between the main lens and the LC microlens is n+p, the height of the viewpoint *A* is H, the position of the center of the (*k* + 1)th microlens is $p_{k+1}$, the offset of the (*k* + 1)th microlens is $\Delta_{k+1}$, and the position of the *k*th element is $h_{k}$. According to the similar triangle,

(13) $$\frac{\Delta_{k+1}}{H-h_{k}}=\frac{g}{n+p}.$$

In addition, the offset of (*k* + 1)th microlens is

(14) $$\Delta_{k+1}=\frac{g}{n+p}(H-h_{k}).$$

Then, the position of the (*k* + 1)th element image is

(15) $$P_{k+1}=h_{k+1}-\Delta_{k+1}.$$

(3) View angle range

For periodicity extraction method, one pixel can be extracted from each element image to obtain a view angle image. Its image resolution is the number of element image. If the number of LC microlens is *N* × *N*, the resolution of the view angel image is *N* × *N*.

After twice imaging, the object has an element images. For every single microlens, there is a corresponding image. According to the similar triangle, the size of the pixels $\Delta P_{k}$ for the *k*th microlens is

(16) $$\frac{\Delta P_{k}}{\left\|AB\right\|}=\frac{q}{n+p}.$$

However, for aperiodicity extraction method, the extracted point changes to the extracted block $\Delta P_{k}$. Thus, the resolution of the view angle image has altered to $\Delta P_{k}^{2}N^{2}$. In this way, the view resolution has been improved $\Delta P_{k}^{2}$ times compared to the periodicity extraction method [Code S1].

For periodicity extraction method, the view angle range $\left[-\theta_{1},\theta_{1}\right]$ is

(17) $$\tan\theta_{1}=\frac{r_{LC}}{g}.$$

For aperiodicity extraction method, the view angle range $\left[-\theta_{2},\theta_{2}\right]$ is

(18) $$\tan\theta_{2}=\frac{r_{LC}-\Delta P}{g}=\frac{r_{LC}-\frac{q}{n+p}\left\|AB\right\|}{g}=\frac{\left(n+p)r_{LC}-q\left\|AB\right\|\right)}{g(n+p)}.$$

(4) High resolution light field imaging

At 0 Vrms (root-mean-square, rms), LC microlens is not activation state. In the light field imaging system, the LC microlens without operation state does not affect final imaging. Acquired 2D image at this state has a full resolution at sensor level, expressed as *I*_0_. When the external electric field adjusts to a voltage value, the activated LC microlens would operate normally. At this moment, the light field imaging can be formed, as shown in Figure 6. And the extracting view image can be expresses as *I*_1_.

The high resolution imaging generating processing is as follows. Firstly, in order to remove the noise, the view angle image is processed by a bilateral filter. It can preserve the edge information. Then, the image is sharpened by Laplace operator. Finally, the weighted average method is utilized:

(19) $$I_{k}=\frac{\sum_{k=0,1}I_{k}\omega_{k}}{\sum_{k=0,1}\omega_{k}}.$$

where $\omega_{k}$ represents the weighted value. The weight value for every image is defined as the coefficient of Laplacian transform and image gradient [Code S1].

## 3. Results

### 3.1. LC Microlens Doped with MWCNTs

**Figure 7:**
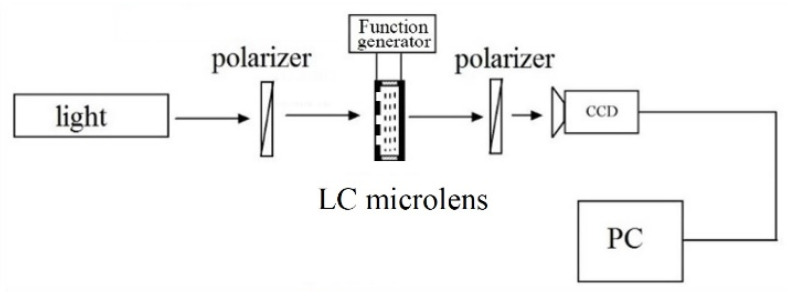
Experimental setup for measuring the classic electro-optical features of the proposed LC microlens.

In Figure 7, a CCD with 10 million pixels, 1/2.3 inch, and 6.4 mm × 4.8 mm in size, from Mindvision Co. (Shenzhen, China), has been utilized. The resolution of the CCD is about 3664 × 2748, and the pixel pitch is about 1.6 μm. Every single LC microlens corresponds to a sub-CCD of approximately 100 × 100 on the imaging plane. The rubbing direction of the LC lens has a 45 degree with a polarizer. The LC lens has been driven by a function generator with a tunable root-mean-square (rms) applied voltage at 1 kHz frequency. During the whole measurement, both the LC lens has been fixed without any mechanical movements. The distance between the main lens and LC microlens is about 1.1 mm.

**Figure 8:**
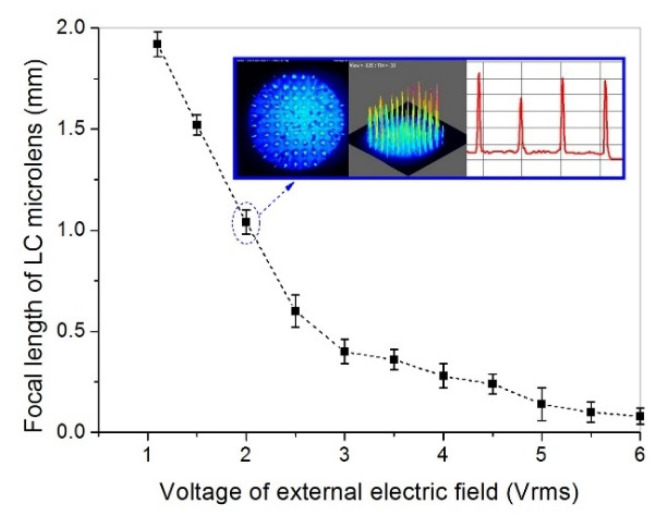
Relationship between the focal length of the LC microlens and the voltage of the external electric field loaded on the LC microlens. The built-in diagram is the 2D image, 3D image, and point spread function (PSF) of the LC microlens under the voltage of 2.0 Vrms.

To evaluate the classic electro-optical performances of the proposed LC microlens, the range of electronically tunable focal length are firstly measured. In consideration of LC birefringence, the tunable focusing properties are the major advantage of the LC microlens. The focal length equation of the LC microlens is $f=r_{LC}^{2}/\left(2\Delta n\cdot d_{LC}\right)$, where $r_{LC}$ means diameter of the circular electrode pattern on the top substrate, Δn is the refractive index difference between the center area and the margin area in LC layer, and $d_{LC}$ is the thickness of the LC layer [14]. The white light is the source. To measure the focal length of the LC microlens, United States Air Force (USAF) 1951 has set in front of CCD. A spatial resolution of 32.0 lp/mm has chosen in the resolution chart in order to measure its image details. When the applied voltage of the LC microlens has changed to a value, the distance between the LC microlens and the imaging sensor has been immediately adjusted until the image of the resolution chart is clear again. With this subjective judgment method, the focal lengths of the LC microlens at different applied voltages have been recorded. The measured focal length of the LC microlens as a function voltage of the external electric field is presented in Figure 8. The relationship between the voltage of the external electric field and the focal length has an inverse proportion. When the external applied voltage is adjusted from 0 Vrms to 6.0 Vrms, the focal length of the LC microlens is capable of switching from 0.08 mm to 1.92 mm. Compared to the conventional LC microlens (the same structure, especially the LC microlens without dopant, the tunable focal length range just from 0.06 mm to 1.8 mm), the doped dopant has improvements on the focal length range of the LC microlens. For the proposed LC microlens, the nematic LC and added MWCNTs under the external electric field can form charge transfer complex by electrostatic force. Compared to the pure LC microlens, the formed charge transfer complex just needs a little force to rotate [35]. In addition, metal property of MWCNTs could also help LC molecular efficiently align in order under the external electric field. With the dopant of those nanoparticles, the range of focal length can be extended in a certain extent. In the built-in image in Figure 8, there are 2D image, 3D image and corresponding PSF of the LC microlens at 2.0 Vrms. Those 2D image and 3D image have been measured by a beam quality analyzer from Dataray Co. From those acquired data, the LC microlens has good beam consistency between neighbor microlenses.

To present the response time of the LC microlens doped with MWCNTs, the response time from one imaging state to another imaging state has been measured. Table 1 presents the compared results between the conventional LC microlens and the proposed LC microlens under the same classic sandwich structure. Although the response time is dependent on many factors, the thickness of the LC layer is still the most important factor. For LC microlens, the thickness of the LC layer is usually thin compared to the LC lens. Thus, the response time of LC microlens is relatively faster than the LC lens. But, the current LC microlens still has room to improve this property. Without doped MWCNTs, the switching time of the conventional LC microlens is 0.19 s. When the MWCNTs have been doped, the response time can reduce to 0.056 s. This means the dopant can shorten the response time of the LC microlens. The switching time is very important for the proposed method for obtaining high resolution of light field imaging. For measuring the operation time of LC microlens, the starting time and the end time have been defined as the time points when the transmitted light power has increased to 10% and 90% of the initial value under an external applied voltage respectively. The results show that the proposed LC microlens has shortened its response time and has been driven by a relatively low operation voltage compared to the conventional one. Overall, the proposed LC microlens dramatically improves about 40% of the operation time and reduces about 70% of the operation voltage. The reason for improvement is that the charge transfer complex formed by LC and MWCNTs can rotate with a little force under the same external electric field [35]. In this way, the LC microlens doped with MWCNTs could have faster response time because of easily rotating under the same external conditions.

### 3.2. High Resolution Light Field Imaging

**Figure 9:**
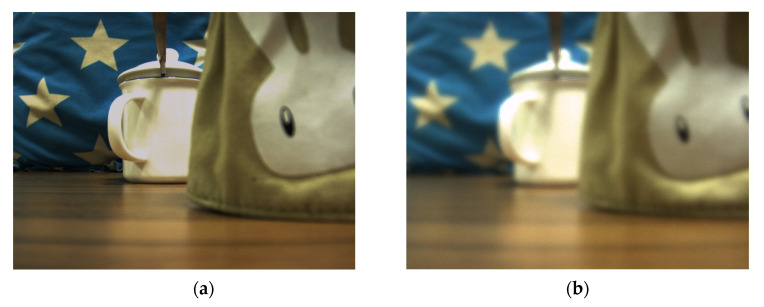
The light field imaging based on the proposed LC microlens under different voltages of the external electric field: (**a**) is at 0 Vrms; (**b**) is at 2.0 Vrms.

The basic optical configuration, as outlined in the operation principle section, comprises a main lens with a 25 mm fixed focal length, a LC microlens, and a CCD in Figure 2. The main lens is utilized to focus on an object of interest at a desired depth. The light transmits the object, then it can be converged by the LC microlens. The transmitted light finally falls on the CCD. Thus, the light field imaging can be formed. In measurement, a white cup has been 1500 mm away from the LC microlens as an object. Then, a bag with a white rabbit icon has set in the front of the object, 1300 mm away from the LC microlens. In the scene, an auxiliary light for the objects has been utilized. For the conventional light field imaging, the periodicity extraction method is usually adopted. The view angle image resolution is just the number of microlens. If the number of microlens is *N* × *N*, the extraction view angle image resolution is also *N* × *N*. As the light field imaging system consists of microlens and CCD, the number of microlens is a constant. That is the reason why the conventional periodicity extraction method has relatively low resolution for view angle image. To solve this problem, the LC microlens is introduced to replace the conventional microlens. As it is well-known, the LC microlens can be tunable by the external electric field. With the use of the above mentioned experimental setup in Figure 2, there are only two objects in the scene. At 0 Vrms, the LC microlens is not activated. All the light pass through the LC microlens. It has no affection for the final imaging. In Figure 9a, the result under this condition has been presented. The resolution of this condition is as high as the full resolution of CCD. But, this image is just the conventional acquisition 2D image without recording any direction information of incident light. Then, the applied voltage across LC microlens has been adjusted to 2.0 Vrms. Under this situation, the LC microlens has been activated. The gradient refractive index array has formed in the LC layer. With the use of dopant, this proposed LC microlens has a relatively good performance on response time, which is very fit for applying in light field imaging. The light field data with recording the direction of the incident light has obtained. Compared to the state at 0 Vrms, the presented result is quite different. The most intuitive expression is many ring-like patterns at the edge of the object in the image, as shown in Figure 9b. Owing to be 2D slice of the light field, the presented image only shows the horizontal and vertical dimensional information. In fact, those ring-like pattern have recorded the direction information of incident light. In other words, the patterns have been overlaid light information in all directions. At the later section, this phenomenon will be further discussed.

**Figure 10:**
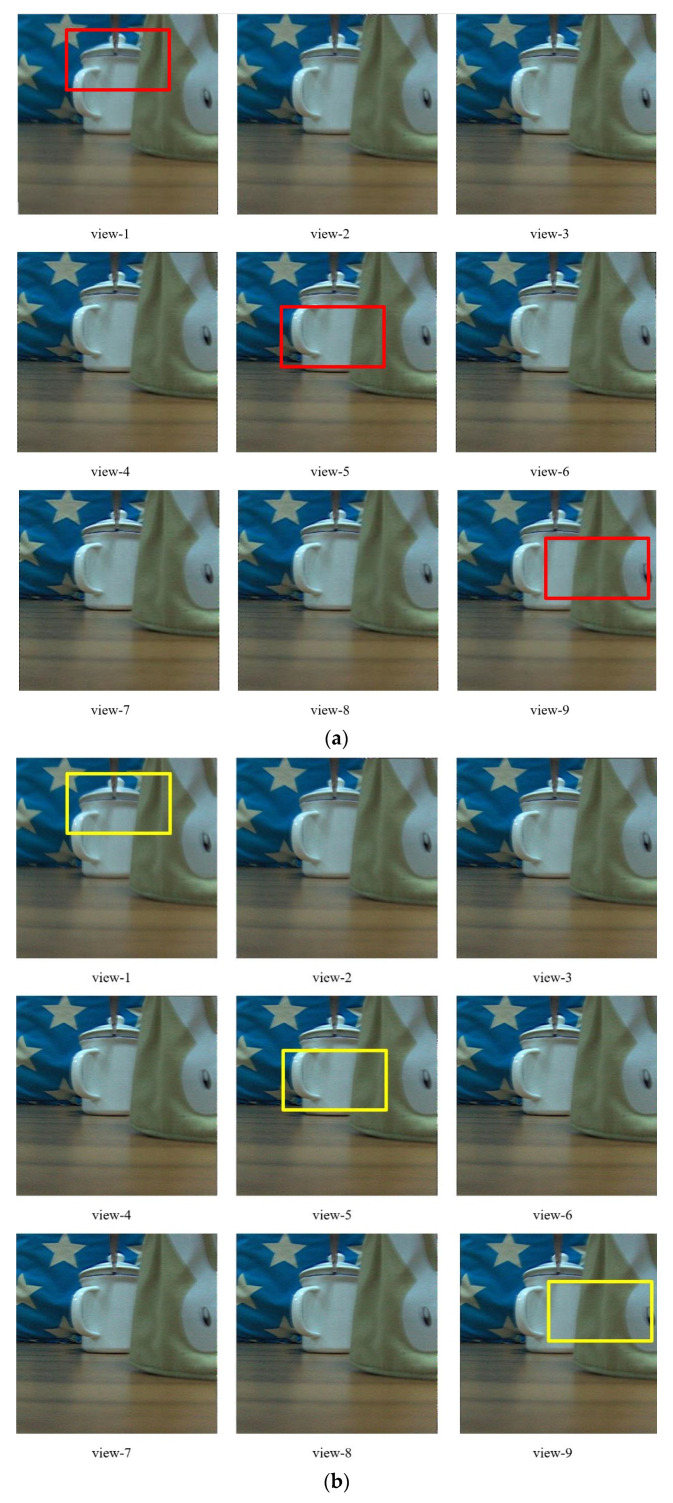

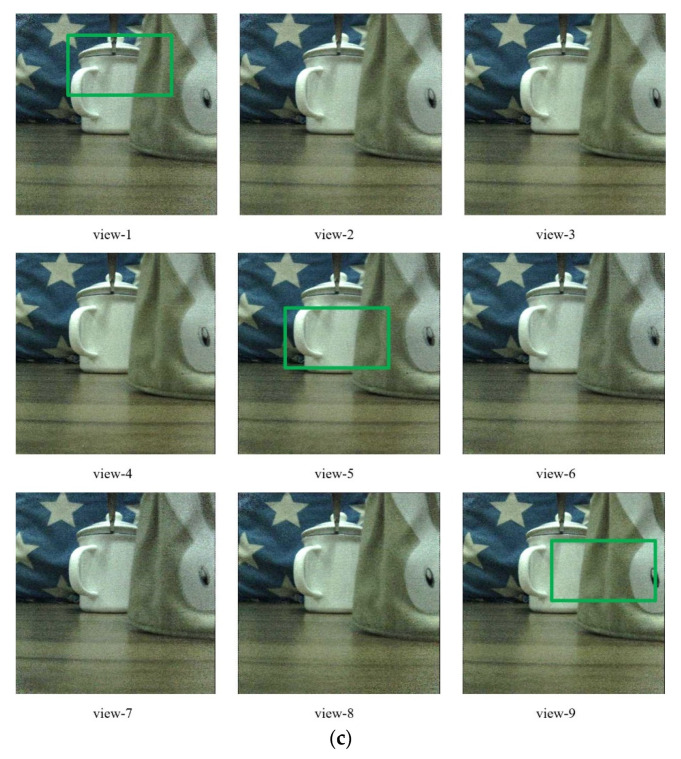
Comparative result images. (**a**) Extracted view angle image based on the proposed aperiodicity method with the proposed LC microlens; (**b**) the corresponding weighted average result images of (**a**); (**c**) extracted view angle image based on the conventional periodicity method with the conventional LC microlens. To present much more details, those images have been magnified to a certain extent.

For the conventional light field, the different view angle image is acquired by the periodicity extraction method from the light field data. A pixel from the same position of every elemental image has been chosen, then they have tiled together to form a view angle image. For different view angle image, the result image can be got by changing the relative position in periodicity extraction. However, this method could result in some problems. As the pixel has chosen by the same position in the element image, the situation is so special that all chosen pixels have just focused on the same object plane. In fact, the pixels should be from different object planes. On the other hand, the one pixel to form a view angle image would have a clear trace of splicing. That will cause unnatural image. At present, some complicated algorithms have been proposed to solve this issue. The process is needed a very huge calculation. In some extreme cases, it would limit the light field imaging application. In this study, a relatively simple method is given in Section 2. With the aperiodicity extraction process, different view angle images can be obtained. To present intuitively and accurately, nine views have been presented. The various factors, such as extracted time, image quality, and block of pixels effect, are all needed by comprehensive consideration. Thus, the number of nine is a compromise value. In the aperiodicity extraction, 3 × 3 pixels are chosen as the block. Hence, the view resolution is 384 × 384. Figure 10a show the different view angle image based on the proposed extraction method via the proposed LC microlens. Compared with Figure 10c, the view resolution can improve about nine times. In the macro-perspective, there are differences between those results. In some detail, view-1 has a focus on the lid of the white cup, view-5 has a focus on the body of the white cup, and view-9 has a focus on the white rabbit of the bag. For calculation, CPU i3-2100 and 8 G RAM have been utilized. The time to acquire the final result is about 450 s. Averagely, the extraction time for every view angle image is about 50 s. That is relatively fast algorithm to extract view angle image. Moreover, Figure 10b presents the corresponding weighted average result images, which respectively blends with 0 Vrms image at different view angle images. Compared to the images in Figure 10a,b, the resolution of the latter is much higher than the former. The weighted average algorithm could effectively improve the resolution of light field imaging. Considering the characteristics of those image at 0 Vrms and 2.0 Vrms, the method of blending image at 0 Vrms with the image at 2 Vrms can realize high resolution of light field imaging. The time for switching one state to another state of LC microlens should be taken into account. Because of the dopant, the response time of LC microlens is much faster than the conventional LC one. The value of the extra time is not very large compared to that of Figure 10a, just 0.056 s. For comparison, the results with the periodicity extraction method have also been presented, as shown in Figure 10c. The cost time is about 540 s. For every single image, it requires about 60 s. The extra switching time is 0.19 s with the conventional LC microlens. The view resolution of Figure 10c is just 128 × 128, the same as the number of LC microlens.

## 4. Discussion

**Figure 11:**
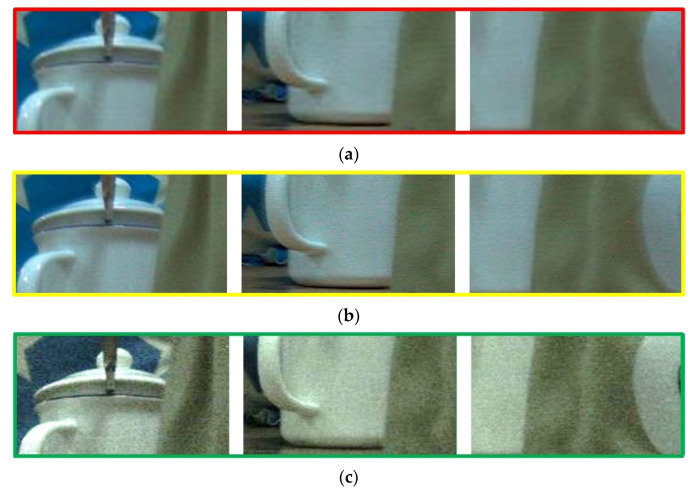

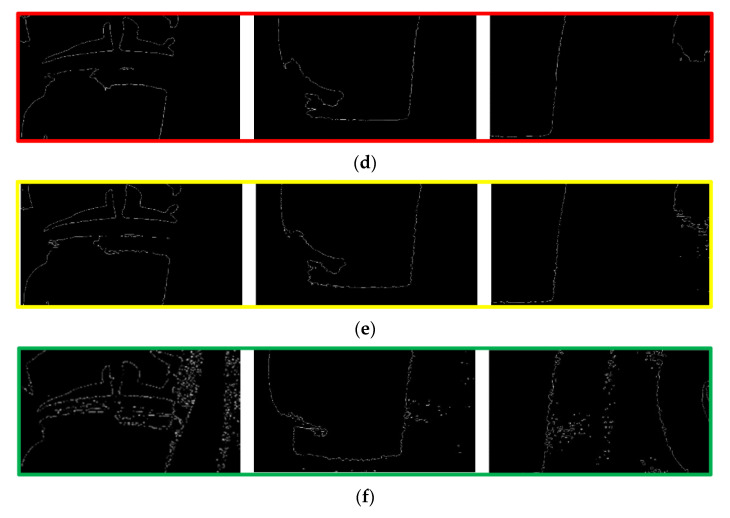
Comparison results of the reconstructed view images. (**a**) The enlarged image of Figure 10a; (**b**) the enlarged image of Figure 10b; (**c**) the enlarged image of Figure 10c; (**d**) the outline extraction of (**a**); (**e**) the outline extraction of (**b**); (**f**) the outline extraction of (**c**).

For further discussion, the details information in Figure 10 are shown in Figure 11. There are three different color square boxes in Figure 10 to provide several local comparative details. In order to compare intuitively, those results are all magnified five times in Figure 11. Improvement of resolution with the proposed method via the proposed LC microlens is relatively remarkable, as shown in Figure 11a,d. The worst resolutions are Figure 11c,f with the conventional periodicity extraction method via the conventional LC microlens. There is a distinct sense of granularity. In addition, the edges in Figure 11c,f have distinct sawtooth, which can prove that the differences between the comparative results are caused by the extraction method. Therefore, the periodicity extraction method can be seen as the root of the low resolution common problem for the conventional light field imaging. Compared with Figure 11c,f, Figure 11a,d have much better resolution, the granular sensation decreases a lot, and the edges are smoother. Figure 11b,e show the corresponding weighted average result images at different view angle image. With the use of weighted average method, the resolution can be improved in a certain extend.

In order to analyze the image quality of the extracted view angle images, five kinds of unreferenced image quality evaluation functions have been introduced to evaluate the result images calculated by the two extraction methods. The non-reference image quality evaluation functions include Brenner gradient function, Tenengrad gradient function, Standard mean difference function (SMD), Standard mean difference 2nd function (SMD2), and Energy function. Two extraction methods via the proposed LC microlens, aperiodicity and periodicity, are used to extract the view angle image. The result image are quantized and analyzed with the non-reference image quality evaluation function. Table 2 shows the compared experimental results. Those three groups correspond to left image, middle image, and right image, as shown in Figure 11a,c. It can be seen from Table 2 that the view angle image with the use of aperiodicity method has much higher image quality compared to the periodicity method. And the weighted average method has the improvement values compared to the aperiodicity method in a certain extend.

It can be seen from the experimental results that the image quality extracted by the aperiodicity method is much better than that of the periodic method, which shows that the former method is more accurate in positioning pixel points. In terms of presenting the comparative results, the five different image quality evaluation functions are utilized. As a result, the aperiodicity method has improved the resolution of view angle image. On the premise of ensuring the accuracy of the data, when extracting the pixel block with the size of $\Delta P_{k}^{2}N^{2}$, the resolution of the view angle image can be increased by $\Delta P_{k}^{2}$ times. The upper limit of $\Delta P_{k}$ is three times. Otherwise, the block effect will occur. The experimental results show that this method can greatly improve the image resolution of view angle image.

Light field imaging breaks through the limitation that traditional optical imaging only records light intensity, and greatly expands the application scope of optical imaging applications. The smart imaging sensor which combines algorithm with hardware has become a trend in recent years. In this study, the light field imaging system based on LC microlens has been studied. Based on LC microlens sampling principle and geometrical optics, a method combining with aperiodicity extraction of view angle image and the weighted average algorithm has been proposed. This method can take into account in both the resolution of the extracted view image and the accuracy of the data.

## 5. Conclusions

A relatively method to improve the image resolution of view angle image based on LC microlens has been proposed. The quantitatively measurements of LC microlens and the extracted view angle image have also been given. Compared with the conventional periodicity extraction method, the proposed method can solve the common problem in the conventional light field imaging. According to the theoretical analysis and experiments, LC microlens doped with MWCNTs is very suitable to apply in the light field imaging. As it has much faster response time, the weighted average method can be utilized in a relatively short time. With the use of the LC microlens, the improvement for the resolution of view angle image becomes possible. At 0 Vrms, the obtained image has a full resolution of CCD without activation of LC microlens. At 2.0 Vrms, the different view angle images can be extracted according to the obtained light field data. The proposed method can effectively combine the both advantages of them. The LC microlens is the key element device to connect those images at the two states.

However, there still exist some problems. The stability of the LC microlens is needed to concern. As the MWCNT is doped to the nematic LC, the agglomerate phenomenon is a common problem in composite material field. Even if using the grinding-shaking method, those doped MWCNTs will still reunite together one month later. It cannot be compared with other chemical grafting methods on the stability. In addition, there are still some noises in the final view angle images. In this study, Laplace operator was utilized to reduce those noises. But, there is still room to improve the image quality in future study.

## Figures and Tables

**Table 1:** Comparison results among two types LC microlens.

Samples	Applied Voltage (Vrms)	Focusing Time ^1^ (s)
The conventional LC microlens with the same structure	~3.6 Vrms	0.19 s
The proposed LC microlens doped with MWCNTs	~2.0 Vrms	0.056 s

^1^ The focusing time is measured for several times, and the final result is an average.

**Table 2:** Image quality comparison result.

Group	Methods ^1^	Brenner	Tenengrad	SMD	SMD2	Energy
1	Aperiodicity	13.3598	531.2426	27.4526	2.1687	14.3888
	Weighted average	26.0006	533.3033	55.3037	2.9036	15.9905
	Periodicity	8.9171	527.6577	20.7732	1.5732	9.9713
2	Aperiodicity	24.1978	528.2426	42.4632	7.8036	33.2994
	Weighted average	45.3598	529.0915	97.2692	8.2571	97.2692
	Periodicity	20.6397	528.9765	21.7168	2.7442	13.0914
3	Aperiodicity	3.3625	531.2426	17.7065	5.7895	4.4805
	Weighted average	16.1247	532.0854	42.6130	6.3879	8.4805
	Periodicity	0.8503	525.0854	8.7082	1.7161	2.8505

^1^ In order to present clearly, the LC microlens with aperiodicity method is the proposed one, and the LC microlens with periodicity method is the conventional one.

## Supplementary Materials

The following are available online at https://www.mdpi.com/1424-8220/20/19/5557/s1, Code S1: core algorithm of aperiodicity extraction and core algorithm of weighted average.
